# Identification of newborns at risk for autism using electronic medical records and machine learning

**DOI:** 10.1192/j.eurpsy.2020.17

**Published:** 2020-02-26

**Authors:** Rayees Rahman, Arad Kodesh, Stephen Z. Levine, Sven Sandin, Abraham Reichenberg, Avner Schlessinger

**Affiliations:** 1 Department of Pharmacological Sciences, Icahn School of Medicine at Mount Sinai, New York, New York, USA; 2 Department of Mental Health, Meuhedet Health Services, Tel Aviv, Israel; 3 Department of Community Health, University of Haifa, Haifa, Israel; 4 Department of Psychiatry, Icahn School of Medicine at Mount Sinai, New York, New York, USA; 5 Seaver Center for Autism Research and Treatment, Icahn School of Medicine at Mount Sinai, New York, New York, USA; 6 MINDICH Child Health and Development Institute, Icahn School of Medicine at Mount Sinai, New York, New York, USA; 7 Department of Environmental Medicine and Public Health, Icahn School of Medicine at Mount Sinai, New York, New York, USA

**Keywords:** autism spectrum disorder, electronic biomarker, random forest, pharmacology, risk prediction

## Abstract

**Background.:**

Current approaches for early identification of individuals at high risk for autism spectrum disorder (ASD) in the general population are limited, and most ASD patients are not identified until after the age of 4. This is despite substantial evidence suggesting that early diagnosis and intervention improves developmental course and outcome. The aim of the current study was to test the ability of machine learning (ML) models applied to electronic medical records (EMRs) to predict ASD early in life, in a general population sample.

**Methods.:**

We used EMR data from a single Israeli Health Maintenance Organization, including EMR information for parents of 1,397 ASD children (ICD-9/10) and 94,741 non-ASD children born between January 1st, 1997 and December 31st, 2008. Routinely available parental sociodemographic information, parental medical histories, and prescribed medications data were used to generate features to train various ML algorithms, including multivariate logistic regression, artificial neural networks, and random forest. Prediction performance was evaluated with 10-fold cross-validation by computing the area under the receiver operating characteristic curve (AUC; C-statistic), sensitivity, specificity, accuracy, false positive rate, and precision (positive predictive value [PPV]).

**Results.:**

All ML models tested had similar performance. The average performance across all models had C-statistic of 0.709, sensitivity of 29.93%, specificity of 98.18%, accuracy of 95.62%, false positive rate of 1.81%, and PPV of 43.35% for predicting ASD in this dataset.

**Conclusions.:**

We conclude that ML algorithms combined with EMR capture early life ASD risk as well as reveal previously unknown features to be associated with ASD-risk. Such approaches may be able to enhance the ability for accurate and efficient early detection of ASD in large populations of children.

## Introduction

Autism spectrum disorder (ASD) is a neurodevelopmental disorder characterized by impairments in social communication and restricted stereotyped behaviors [1]. Between 2000 and 2014, the number of children with ASD more than doubled, and it is now estimated that ASD affects about 1 in 59 children in the United States [1]. The diagnosis of ASD typically relies on the observation of behavioral symptoms. Although these behaviors manifest at an early age (~1 year), in the overwhelming majority of children, the diagnosis is not ascertained until after the age of 2 [2]. This points to an important challenge because mounting evidence indicates that early diagnosis and interventions improve the outcome for affected children [3,4].

Existing studies have shown that ASD is highly heritable [5]. However, at present, genetic screening cannot reliably predict ASD. For example, despite progress in identifying rare genetic variants associated with ASD, single gene disorders only account for 3–7% of all ASD cases [6]. Thus, unlike other single-gene disorders, such as Huntington’s disease, genetic screening has limited utility in families with idiopathic ASD. In addition, due to the phenotypic heterogeneity of ASD, identification of reproducible genetic variants with significant associations to ASD incidence remains challenging [7]. Furthermore, even the strongest known ASD-risk factor, a sibling with ASD, is not useful in more than 95% of ASD cases—because there is no older sibling diagnosed with ASD before the case is born [8]. Taken together, risk-assessment models that are based on genetic information alone do not perform reliably in the context of the complex etiology of ASD.

The overwhelming majority of studies into nongenetic ASD-risk factors typically consider only one risk-factor in isolation, for example, paternal age [9] or antidepressant use during pregnancy [10]. However, individual features are not sufficiently accurate in predicting individual risk [10–12]. Furthermore, such measures of risk were often derived from studies that used traditional statistical methods to identify risk factors. Traditional statistical approaches are limited in their capacity to combine potentially predictive features [13]. The effective combination of ASD-prevalence in the family, prescription drugs, and socioeconomic variables may yield greater predictive power than one factor alone.

Machine learning (ML) provides an approach to efficiently combine features derived from large data, thereby generating clinically-relevant predictions [13–15]. Studies based on analysis of electronic medical records (EMRs) and application of ML have discovered complex relationships among various features and disease risk, from genomic analysis to activities in the emergency room [13–16], but, to the best of our knowledge, has not yet been applied to address the ASD epidemic.

The aim of the current study was to test the ability of ML models applied to EMRs to predict ASD early in life. To address our aim, we tested the ability of an array of parental characteristics available to predict the risk of ASD in offspring in a large population-based sample.

## Methods

### Study approval

This study was approved by the Institutional Review Board at the University of Haifa and the Helsinki Ethics Committee at Meuhedet healthcare. Those bodies waived the need for informed consent because the study data were deidentified. This study followed the Standards for Reporting of Diagnostic Accuracy (STARD) and the Transparent Reporting of a Multivariable Prediction Model for Individual Prognosis or Diagnosis reporting guidelines [17,18].

### Data source

EMR data were obtained from a population-based case–control cohort study ascertained through a large health maintenance organization in Israel (Meuhedet) [19]. All Israeli citizens are required to purchase a medical insurance plan from one of several health maintenance organizations, which offer equivalent medical provision and fees, limiting potential selection bias in our study. Details of the ascertainment and source population, as well as the representativeness of the cohort have been previously reported [19]. The Meuhedet cohort used in this study includes EMR data on children born in Israel from January 1, 1997 through December 31, 2007, and their parents. Children were followed up for ASD diagnosis from birth to January 26, 2015. The analytic sample consisted of 1,397 ASD cases across 1,207 father–mother pairs and 94,741 controls across 34,912 mother–father pairs.

### Validity of ASD diagnosis

ASD diagnosis followed the International Classification of Diseases, Ninth Revision (ICD-9) and International Statistical Classification of Diseases and Related Health Problems, Tenth Revision (ICD-10). All children with suspected ASD underwent evaluation by a panel of social workers, a psychologist, and one of a trained psychiatrist, a developmental behavioral pediatrician, or a child neurologist. The final diagnosis was made by a board-certified developmental behavioral pediatrician.

### Data preparation

Parental EMR data were selected where all nondrug treatments, such as medical devices, were removed along with rows containing non-UTF8 formatted data. Next, a set of Anatomic Therapeutic Classification (ATC) codes were obtained from the world health organization (WHO; www.whocc.no; 2016 version). Drugs that correspond to multiple ATC classifications were combined into a single ATC code. ATC codes were mapped to drug names present in the data; multiple drug names corresponding to the same ATC or known combinations of drugs were manually annotated with unique ATC codes. Multiple prescriptions corresponding to the same ATC code within a single individual were then filtered out. Although our dataset includes the date the prescription was filled, it does not directly indicate the duration of drug usage.

### Features set used for training

**Figure 1.**
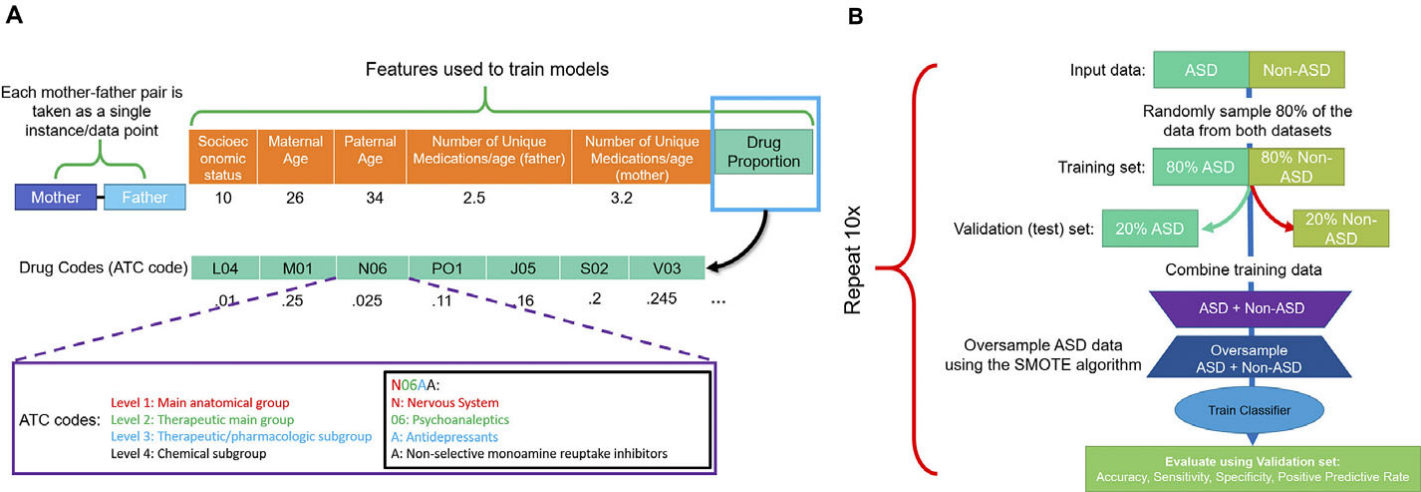
Workflow used to build the machine learning model of autism spectrum disorder (ASD) incidence. To evaluate the utility of electronic medical record (EMR) and machine learning for predicting the risk of having a child with ASD, we developed a comprehensive dataset. (A) For each mother–father pair, the parental age difference, number of unique medications either parent has taken, the socioeconomic status, as well as the proportion of drugs, by level 2 Anatomic Therapeutic Classification (ATC) code, taken by the parent were used for further analysis. (B) Workflow of performing 10-fold cross-validation to evaluate model performance. First, the data were partitioned into ASD and non-ASD cases, where 80% of the data were randomly sampled as training set, and 20% were withheld as testing set. The training set was then combined and the synthetic minority oversampling technique (SMOTE) was used to generate synthetic records of ASD cases. A multilayer perceptron (MLP), also known as feedforward neural network, logistic regression, and random forest models were trained using the oversampled training data. They were then evaluated on the testing data based on sensitivity, precision, sensitivity, false positive rate, and area under the ROC curve (AUC; C-statistic). Since the testing data did not have synthetic cases, the model performance is indicative of performance of real data. This process was repeated 10 times and average model performance was reported.

For each parent, 89 features were selected as predictors to train the different ML models. These features included prescribed medications (86 features) and medical histories (e.g., parental age difference; Figure 1A), as well as sociodemographic characteristics (e.g., age, socioeconomic status) [19]. All noncategorical features were normalized using the Softmax normalization technique (Figure 1A). Missing values were imputed using the rfImpute method of the randomForest package in the R programming language depending on ASD status [20].

## Statistical Analysis

### ML models

Several statistical and ML methods were used to predict child ASD status. We applied logistic regression, artificial neural networks (also known as multilayer perceptron or MLP), and random forest to predict the likelihood of offspring ASD diagnosis based on the parental features described. Notably, one advantage of a decision tree-based learning is that the importance explanatory features can be extracted after training. Random forest, an ensemble decision tree learner, was utilized for its comparable predictive performance to regression-based techniques for EMR-based datasets [14,15].

### Model training and evaluation

To evaluate each model, 10-fold cross-validation was employed (Figure 1B). Eighty percent of all ASD and non-ASD mother–child pairs were sampled from the original dataset, whereas the remaining 20% were kept as the validation set. Due to the significant class imbalance present in the data, the Synthetic Minority Over-sampling Technique (SMOTE) was used to increase the proportion of ASD cases fivefold with the “DMwR” package in the training set, while the validation set sampling was unchanged [21]. Oversampling under-represented data (and under-sampling overrepresented data) in training is an established approach for developing predictive models for ML problems with large class imbalances. This approach has been used to improve the prediction of breast cancer survivability [22] and Alzheimer’s disease susceptibility [23].

After oversampling, the resultant dataset was used to train either the logistic regression model using the “glm” method in the R programming language, the MLP using the R-language interface for the Stuttgart Neural Network Simulator (RSNNS) package, or a random forest model implemented in the R programming language using the “randomForest” package [20]. We performed hyperparameter optimization of the ML algorithms using the caret package [24]. The final MLP model used in 4 hidden layers of 5, 10, 10, and 2 nodes each, under default settings. The final random forest model used 1,000 subtrees and randomly sampled 20 features per tree. The remaining 20% of the ASD and non-ASD data were used as the validation set for the models.

**Figure 2.**
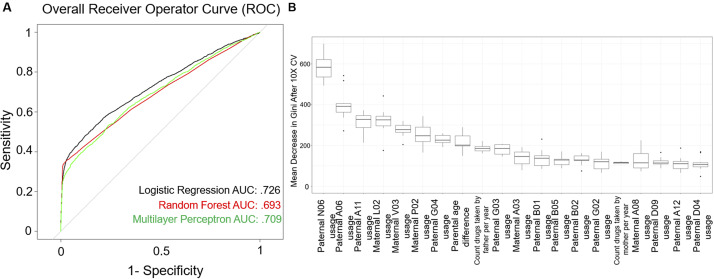
Performance of machine learning-based autism spectrum disorder (ASD)-risk predictor. A balanced dataset was generated to train various algorithms to predict the probability of an ASD child from the electronic medical record of the parents. (A) Receiver operator characteristic (ROC) curves for all methods tested: logistic regression, random forest, and MLP. (B) Boxplot of importance values of each feature in the random forest model after 10-fold cross-validation (10× CV). Importance of a feature is defined as the mean decrease in Gini coefficient when training a model. Level 2 Anatomic Therapeutic Classification (ATC) codes are represented by an alphanumeric three-letter code.


The literature on prediction of health outcomes often focuses on the area under the receiver operating characteristic (ROC) curve (i.e., AUC or C-statistic) rather than the full spectrum of prediction performance [25]. However, a diagnosis of ASD is a rare outcome, and therefore relying on the C-statistic alone may be biased due to either over- or underestimation [25]. For the evaluation of a clinical prediction tool, it has been recommended to report sensitivity, specificity, accuracy, false positive rate, and precision (positive predictive value [PPV]; Figure 2) to provide a more complete picture of the performance characteristics of a specific model. The validation process was repeated 10 times and the average sensitivity, specificity, accuracy, precision, false positive rate, and area under the receiver operator curve (AUC; C-statistic) across all models were computed.

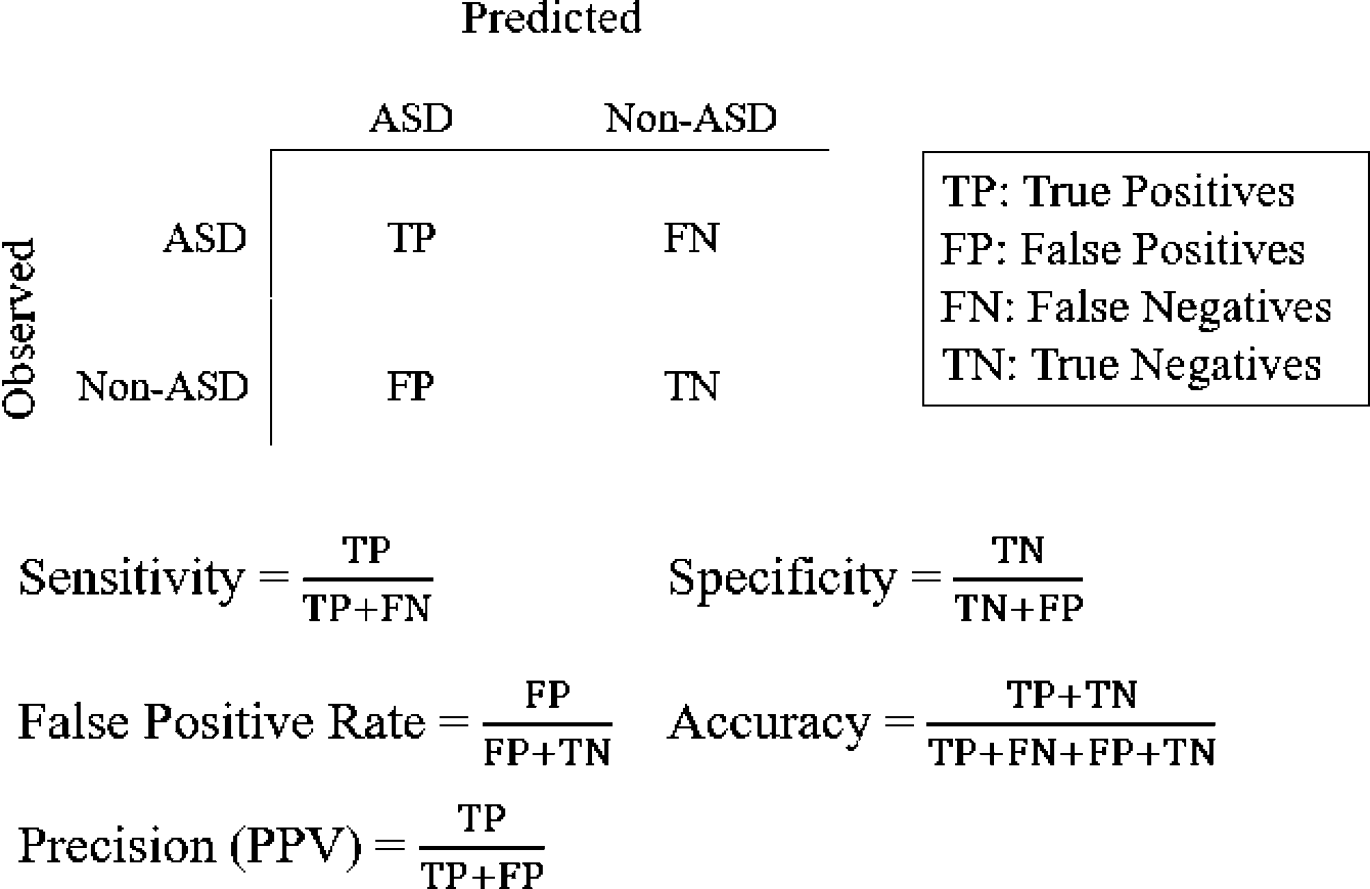
Notably, because oversampling was only utilized during model training while validation was done on unmodified set, the evaluation metrics generated are indicative of classifier accuracy for nonsynthetic cases. As a control, we compared the performance of the model trained on SMOTE generated records to models trained from either down-sampling non-ASD cases or inverse probability class weighting [26,27]. Random forest models trained on SMOTE models (AUC of 0.693; Table 1) outperformed models trained using alternative balanced training approaches (AUC of 0.680 and 0.634, respectively), increasing our confidence in the quality of the SMOTE protocol.

**Table 1.** Performance of classifiers after 10-fold cross-validation

Model	True positives (TP)	False negatives (FN)	False positives (FP)	True negatives (TN)	Sensitivity	Specificity	Precision (PPV)	False positive rate (FPR)	Accuracy	AUC
Logistic regression	88.1	160.7	175.4	6,202	0.354	0.972	0.336	0.0275	0.949	0.727
MLP	74.8	172.7	126.7	6,252	0.301	0.98	0.393	0.0198	0.955	0.709
Random forest	60.5	188.3	45.6	6,332	0.243	0.993	0.572	0.00715	0.965	0.693
Average	74.5	173.9	115.9	6,262	0.299	0.982	0.434	0.018	0.956	0.709

*Note*: Average performance metric of each classifier including logistic regression, multilayer perceptron (MLP), and random forest tested after 10-fold cross-validation. Additionally, average marks the average performance across all methods.

### Determining importance of features

Gini impurity criterion was applied to determine the relative importance of individual features. The Gini impurity is the probability of an unseen case being incorrectly classified for a given decision or rule. Features with high Gini impurity (or low Gini importance) split the data into impure categories, while features that decrease Gini impurity are able to partition the data into purer classes with larger members. Thus, features with large mean decreases in Gini rank higher in importance for the model. The importance of a feature is defined as the mean decrease in the Gini impurity based on the Random Forest model.

## Results

### Prediction accuracy

After 10-fold cross-validation of the testing dataset, we observed that all models tested had overall similar performance (Table 1, Figure 2). The random forest algorithm classified predicted fewer true positive ASD cases, on average, than logistic regression or the MLP; however, it also predicted far fewer false positives (FPs) compared with logistic regression or MLP, leading to a higher overall PPV (precision) compared with either of the two models. Although logistic regression outperformed both random forest and MLP in terms of AUC, it was the lowest performer in terms of PPV. Finally, MLP generated middling results, performing in-between logistic regression and random forest across all metrics. Overall, the average performance across all models had an accuracy of 95.62%, sensitivity of 29.93%, specificity of 98.18%, PPV of 43.35%, area under the ROC curve (AUC or C-statistic) of 0.709, and false positive rate of 1.81% for predicting ASD in this dataset (Table 1. Figure 2A,B).

### Importance of features

The random forest model allows the identification of top features that enable case classification (i.e., ASD vs. non-ASD) within the multivariate model. Figure 2B shows the top 20 features ranked by median variable importance after 10-fold cross-validation. Top features included parental age differences, and parental number of medications per year, as well as specific maternal and paternal exposure to medications. These include paternal psychoanaleptics, drugs for the treatment of blood conditions, antiparasitic medications, medications for genitourinary system and reproductive hormones, as well as nutritive supplements, maternal endocrine therapies, antheleminitics, gastrointestinal drugs, and antiobesity preparations. Supplemental Tables S1 and S2 present rates of exposure to the ATC-derived features in parents of ASD and non-ASD children.

### Sensitivity analysis

**Figure 3.**
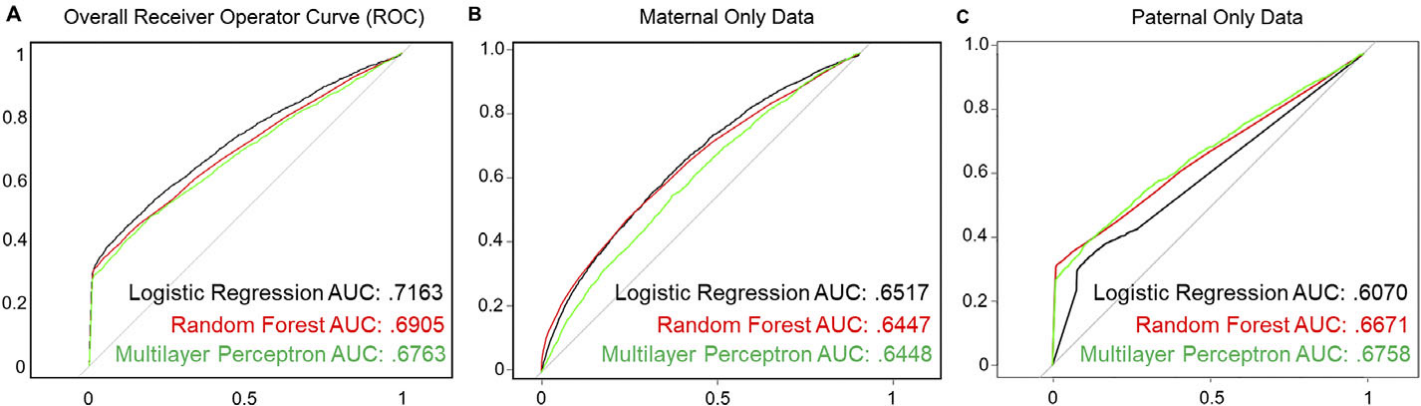
Sensitivity analysis of the generated machine learning models. (A) Receiver operator characteristic (ROC) curves for all methods tested with “missing parental information” label included. (B) ROC curves of models generated when all parental medication data are removed. (C) ROC curves of models generated when all maternal medication data are removed.

ML models are often prone to fitting to, or “memorizing,” specific features or training examples, which can cause models to have poor performance for novel samples; this is called model overfitting. Additionally, imputation of missing data can lead to a potential source of bias in our data. Since any missing features were imputed in our training data, we investigated whether our models were overfitting due to missing information. We included an additional feature labeling cases with missing parental information in order to observe the effect missing data had on predictive performance. The models were then regenerated and evaluated. The models perform similarly, in terms of AUC with or without the feature (Figure 3A and Table 2). This indicates that our models are unlikely to be overfitting to the lack of parental information. Notably, we observe a decrease in the average number of false positives in the logistic regression model, increasing its overall PPV from 33.5 to 43.6% (Table 2). The random forest model, however, has decreased PPV performance, from 57.2 to 52.9%. Finally, we also regenerated models removing features derived from either all paternal or maternal medication history (Figures 3B,C). These models have lower C-statistic compared with those of models integrating both data sources, indicating that EMR records from either parent may explain only part of the risk of ASD in the offspring.

**Table 2.** Performance of classifiers in sensitivity analysis

Model	True positives (TP)	False negatives (FN)	False positives (FP)	True negatives (TN)	Sensitivity	Specificity	Precision (PPV)	False positive rate (FPR)	Accuracy	AUC
Logistic regression	75.7	166	97.9	6,286	0.313	0.985	0.436	0.0153	0.96	0.716
MLP	77.1	167.9	116	6,265.5	0.314	0.982	0.404	0.0181	0.957	0.694
Random forest	57.1	184.6	52	6,332.3	0.236	0.992	0.529	0.0081	0.96	0.687
Average	69.9	172.8	88.6	6,294.7	0.287	0.986	0.456	0.0138	0.959	0.699

*Note*: Average performance metric of each classifier including logistic regression, multilayer perceptron (MLP), and random forest tested after 10-fold cross-validation with “missing parental information” label included. Additionally, average marks the average performance across all methods.

## Discussion

This study of population-based representative data shows that ML applied to EMRs could potentially identify a large proportion of future ASD cases. In the sample studied here, almost one-third of ASD cases could be predicted based on demographic and medical characteristics of their mothers and fathers. The ML models achieved high predictive performance using only routinely collected data in EMRs and without molecular genetic screening. To the best of our knowledge, this is the first study that applied ML on EMR data to specifically predict ASD in a large population of children.

Unlike sensitivity and specificity, which are test properties, precision estimates are affected by the rate of the outcome in the population. Low rates of ASD could lead to low precision and high false-positive rates, even in tests with high sensitivity and specificity, and this, therefore, limits the clinical utility of a prediction algorithm. For example, screening for trisomy 21 in 20- to 30-year-old women (prevalence of approximately 1:1,200) [28], has a precision of only 1.7% with a test with sensitivity higher than 99% and specificity higher than 95% [28]. Despite the modest sensitivity in this study (30%), the specificity was very high (98%), and the precision in the study was acceptable (43%). Thus, our approach may allow high accuracy in identifying patients. To further limit the false positives in this group, additional screening and assessment may be needed.

There are several potential explanations for the gains in the prediction ability by ML. First, ML approaches can identify non-traditional predictors of ASD from complex EMR data. Second, ML methods combine nonlinear interactions between features, which may not be captured by traditional modeling approaches (e.g., logistic regression model) [29]. Third, we applied rigorous approaches to minimize the potential of overfitting of the models.

The features, which had the strongest association with case classification in the ML models, included several previously proposed sociodemographic risk factors for ASD such as differences in parental age [9] and parental number of medications [19]. Interestingly, there were also various medication groups that were associated with ASD classification. We observed a relation with psychoanaleptics, which has a mixed pattern of associations with ASD-risk in previous studies [19,30,31]. We also observed a relation with nutritive supplements and reproductive hormones. Increased and decreased risk of ASD has been previously reported for medications from these groups [32,33]. Maternal metabolic conditions and disruptions in the endocrine system have been associated with ASD-risk [32,33], and contributed to case classification. Taken together, the agreement between our most useful features with previous studies analyzing ASD patients increases the confidence in our combined approach.

Notably, the associations between drugs for the treatment of blood conditions, antiparitic medications, medications for genitourinary system, anthelmintics, as well as gastrointestinal drugs that showed importance for determining ASD-risk warrants further research. Nevertheless, it is important to note that each feature, individually, may not be a good predictor of ASD, as individual features do not, by themselves, separate cases from controls with high precision. As noted above, it is only in the context of the ML algorithm(s) that the combinations of features appear predictive.

Finally, we observed that apparent ASD-risk can only be partially explained using either only maternal or paternal EMR data. Rather, the best predictive performance was obtained by training a model combining both sources of information. This result provides evidence that characterizing ASD incidence requires a multifaceted approach integrating maternal and paternal risk factors.

Our study has several limitations. First, the ML approaches are data driven and, therefore, depend on the accuracy of our data. Although coding errors do occur, the rate of such errors in EMRs has been shown to be very low (rate less than 1%) and accuracy of the data in the Meuhedet health provider is continuously monitored for completeness and accuracy of reporting. Second, the imputation of missing data is a potential source of bias. However, the imputation by random forest is known to be a rigorous technique [34]. Third, the study lacked genetic information, which could provide a mechanistic interpretation of the results as well as improve prediction accuracy. Nonetheless, the objective of the present study was to develop ML-based prediction models that can harness available EMR data. Future studies with genetic linked data are warranted. Finally, while we show associations between several medication groups and ASD, it is important to note that this does not provide evidence that these therapeutics are causally related to ASD. Rather, parental usage of these medications may be indicative of an underlying parental genetic predisposition to ASD, or the modifying role of environmental exposure on genetic predisposition.

The current study demonstrates the feasibility and potential of routinely collected EMR data for the identification of future children at high-risk of ASD. The results also show the potential utility of data driven approaches for uncovering previously unidentified risk factors for ASD. Although certainly not causal or perfect, our results present reason for cautious optimism that recent developments in ML methodologies will be able to enhance the ability for accurate and efficient early detection of ASD in large populations of children, and allow interventions to be targeted to the small number of individuals who are at greatest risk.

## Data Availability

The data that support the findings of this study were made available from Mehuhedet. Restrictions apply to the availability of these data, which were used under license for this study. The code is available on the Schlessinger Laboratory GitHub repository: https://github.com/schlessinger-lab/ASD-ML-EURPSY.

## Supplementary material

For supplementary material accompanying this paper visit https://doi.org/10.1192/j.eurpsy.2020.17. click here to view supplementary material
