# Supplementary material for: Identification of newborns at risk for autism using electronic medical records and machine learning
Source: Eur Psychiatry. 2020 Feb 26;63(1):e22. doi: 10.1192/j.eurpsy.2020.17 (PMC7315872; doi:10.1192/j.eurpsy.2020.17)
Supplement: Supplementary file 1 [file S0924933820000176sup001.docx]

**Supplementary data: Identification of newborns at risk for autism using electronic medical records and machine learning**

**Supplementary Table 1. Rates of exposure of mothers to ATC-derived features included in the ML algorithms.** A two-tailed Fisher’s exact test was used to calculate the association of level 2 ATC codes between mothers of children with ASD and mothers of non-ASD children across the mother’s entire medical record.

| **ATC Code** | **% use in ASD cases** | **% use in non-ASD** | **p-value** |
| --- | --- | --- | --- |
| A01 | 0.934801762 | 0.975763638 | 1.81E-12 |
| A02 | 0.641409692 | 0.650457798 | 0.537829756 |
| A03 | 0.546255507 | 0.557359391 | 0.473831179 |
| A04 | 0.011453744 | 0.016696161 | 0.22004783 |
| A05 | 0.003524229 | 0.003770101 | 1 |
| A06 | 0.51277533 | 0.556589982 | 0.004608153 |
| A07 | 0.923348018 | 0.961529584 | 1.61E-08 |
| A08 | 0.250220264 | 0.203585443 | 0.000282325 |
| A09 | 0.095154185 | 0.0776333 | 0.038834696 |
| A10 | 0.400881057 | 0.433176887 | 0.036240976 |
| A11 | 0.862555066 | 0.913980149 | 4.15E-08 |
| A12 | 0.481057269 | 0.508655844 | 0.077649178 |
| B01 | 0.472246696 | 0.529122105 | 0.000252968 |
| B02 | 0.441409692 | 0.477187043 | 0.021792222 |
| B03 | 0.866960352 | 0.927290913 | 1.76E-11 |
| B05 | 0.792951542 | 0.847041625 | 3.42E-06 |
| C01 | 0.836123348 | 0.863660845 | 0.012042127 |
| C02 | 0.044052863 | 0.038239594 | 0.335125849 |
| C03 | 0.039647577 | 0.036239132 | 0.563090374 |
| C04 | 0.001762115 | 0.001692698 | 1 |
| C05 | 0.925110132 | 0.962606755 | 2.40E-08 |
| C07 | 0.0969163 | 0.09055936 | 0.484209844 |
| C08 | 0.06784141 | 0.064091713 | 0.61393035 |
| C09 | 0.054625551 | 0.050088482 | 0.480003568 |
| C10 | 0.080176211 | 0.086558437 | 0.507677426 |
| D01 | 0.88722467 | 0.93806263 | 1.02E-09 |
| D02 | 0.082819383 | 0.117180888 | 0.00037567 |
| D03 | 0.084581498 | 0.106870816 | 0.017674319 |
| D04 | 0.67753304 | 0.704701085 | 0.057949027 |
| D05 | 0.03876652 | 0.0276987 | 0.040293879 |
| D06 | 0.92246696 | 0.95468185 | 5.95E-06 |
| D07 | 0.943612335 | 0.973455413 | 2.38E-07 |
| D08 | 0.414977974 | 0.43256136 | 0.260620223 |
| D09 | 0.474008811 | 0.513041471 | 0.012139784 |
| D10 | 0.864317181 | 0.910979457 | 8.22E-07 |
| D11 | 0.707488987 | 0.766792337 | 1.06E-05 |
| G01 | 0.909251101 | 0.951988921 | 9.53E-09 |
| G02 | 0.621145374 | 0.702085097 | 2.67E-08 |
| G03 | 0.85814978 | 0.879587597 | 0.037466211 |
| G04 | 0.596475771 | 0.635377395 | 0.010211682 |
| H01 | 0.020264317 | 0.012079711 | 0.025995176 |
| H02 | 0.913656388 | 0.951142571 | 4.03E-07 |
| H03 | 0.082819383 | 0.100484727 | 0.055360695 |
| H04 | 0.001762115 | 0.002385166 | 1 |
| H05 | 0.001762115 | 0.001000231 | 0.342081108 |
| J01 | 0.979735683 | 0.995152728 | 1.79E-07 |
| J02 | 0.592951542 | 0.663229976 | 2.21E-06 |
| J05 | 0.256387665 | 0.254904978 | 0.915186377 |
| J06 | 0.422907489 | 0.449488343 | 0.086924685 |
| J07 | 0.488105727 | 0.49903824 | 0.495931356 |
| L01 | 0.337444934 | 0.371931984 | 0.020951562 |
| L02 | 0.377092511 | 0.342463645 | 0.019112795 |
| L03 | 0.00969163 | 0.008925137 | 0.742727883 |
| L04 | 0.012334802 | 0.01477264 | 0.605548002 |
| M01 | 0.851101322 | 0.895206586 | 1.11E-05 |
| M02 | 0.812334802 | 0.85765946 | 6.41E-05 |
| M03 | 0.246696035 | 0.248826652 | 0.886186502 |
| M04 | 0.011453744 | 0.009540663 | 0.525607681 |
| M05 | 0.007048458 | 0.010540894 | 0.354470195 |
| M09 | 0.037004405 | 0.02862199 | 0.117874254 |
| N01 | 0.669603524 | 0.720704778 | 0.000307682 |
| N02 | 0.84845815 | 0.89189813 | 1.81E-05 |
| N03 | 0.107488987 | 0.082403632 | 0.004487343 |
| N04 | 0.098678414 | 0.084019389 | 0.096058033 |
| N05 | 0.338325991 | 0.307378626 | 0.032187244 |
| N06 | 0.550660793 | 0.527506348 | 0.136742324 |
| N07 | 0.155066079 | 0.181272601 | 0.026471095 |
| P01 | 0.313656388 | 0.302685235 | 0.439025188 |
| P02 | 0.226431718 | 0.214664923 | 0.366393221 |
| P03 | 0.044933921 | 0.052012003 | 0.326814408 |
| R01 | 0.964757709 | 0.987535585 | 1.14E-07 |
| R02 | 0.881057269 | 0.925290452 | 4.79E-07 |
| R03 | 0.851101322 | 0.881511118 | 0.003225105 |
| R05 | 0.817621145 | 0.825190429 | 0.515140506 |
| R06 | 0.793832599 | 0.794106332 | 0.96948946 |
| R07 | 0.377092511 | 0.40940217 | 0.034772558 |
| S01 | 0.982378855 | 0.994768023 | 1.65E-05 |
| S02 | 0.948898678 | 0.979610679 | 4.86E-09 |
| S03 | 0.923348018 | 0.960760175 | 4.87E-08 |
| V01 | 0.206167401 | 0.192044318 | 0.255552885 |
| V03 | 0.433480176 | 0.406016773 | 0.072717905 |
| V04 | 0.037885463 | 0.047318612 | 0.162571372 |
| V06 | 0.001762115 | 0.001692698 | 1 |

**Supplementary Table 2. Rates of exposure of fathers to ATC-derived features included in the ML algorithms.** A two-tailed Fisher’s exact test was used to calculate the association of level 2 ATC codes between fathers of children with ASD and mothers of non-ASD children across the father’s entire medical record.

| **ATC Code** | **% use in ASD cases** | **% use in non-ASD** | **p-value** |
| --- | --- | --- | --- |
| A01 | 0.841428571 | 0.924790438 | 8.48E-13 |
| A02 | 0.571428571 | 0.634042223 | 0.000965221 |
| A03 | 0.284285714 | 0.339723688 | 0.002367702 |
| A04 | 0.002857143 | 0.003182242 | 1 |
| A05 | 0.002857143 | 0.002871779 | 1 |
| A06 | 0.475714286 | 0.517773983 | 0.032651425 |
| A07 | 0.83 | 0.904998448 | 2.25E-09 |
| A08 | 0.165714286 | 0.215538653 | 0.001475666 |
| A09 | 0.09 | 0.095389631 | 0.691454173 |
| A10 | 0.382857143 | 0.42688606 | 0.022743424 |
| A11 | 0.497142857 | 0.563955293 | 0.000573888 |
| A12 | 0.381428571 | 0.428748836 | 0.014929069 |
| B01 | 0.405714286 | 0.484942564 | 4.43E-05 |
| B02 | 0.355714286 | 0.398556349 | 0.023781476 |
| B03 | 0.321428571 | 0.372787954 | 0.006247734 |
| B05 | 0.687142857 | 0.753104626 | 0.000135999 |
| C01 | 0.758571429 | 0.830797889 | 2.19E-06 |
| C02 | 0.027142857 | 0.024837007 | 0.708049963 |
| C03 | 0.044285714 | 0.049829246 | 0.591173196 |
| C04 | 0.004285714 | 0.005122633 | 1 |
| C05 | 0.824285714 | 0.903834213 | 3.49E-10 |
| C07 | 0.077142857 | 0.097873331 | 0.076102261 |
| C08 | 0.05 | 0.053477181 | 0.79524853 |
| C09 | 0.11 | 0.111533685 | 0.950849293 |
| C10 | 0.192857143 | 0.24161751 | 0.003071315 |
| D01 | 0.708571429 | 0.818457001 | 6.44E-12 |
| D02 | 0.042857143 | 0.076606644 | 0.000508166 |
| D03 | 0.027142857 | 0.034150885 | 0.38915029 |
| D04 | 0.53 | 0.617432474 | 5.32E-06 |
| D05 | 0.027142857 | 0.034073269 | 0.388863457 |
| D06 | 0.834285714 | 0.903058057 | 4.50E-08 |
| D07 | 0.885714286 | 0.931232536 | 2.06E-05 |
| D08 | 0.271428571 | 0.287954052 | 0.367740829 |
| D09 | 0.302857143 | 0.370071406 | 0.000286938 |
| D10 | 0.711428571 | 0.813334368 | 2.19E-10 |
| D11 | 0.625714286 | 0.730440857 | 4.92E-09 |
| G01 | 0.67 | 0.835454828 | 3.61E-25 |
| G02 | 0.461428571 | 0.591819311 | 1.72E-11 |
| G03 | 0.365714286 | 0.406162682 | 0.036029668 |
| G04 | 0.487142857 | 0.551769637 | 0.000908025 |
| H01 | 0.012857143 | 0.008304874 | 0.201595502 |
| H02 | 0.787142857 | 0.887069233 | 2.55E-13 |
| H03 | 0.027142857 | 0.022353306 | 0.431355015 |
| H04 | 0.002857143 | 0.003259857 | 1 |
| H05 | 0.001428571 | 0.001086619 | 0.547974299 |
| J01 | 0.955714286 | 0.986029183 | 1.24E-07 |
| J02 | 0.305714286 | 0.388854393 | 9.16E-06 |
| J05 | 0.115714286 | 0.162216703 | 0.000830831 |
| J06 | 0.354285714 | 0.394986029 | 0.031936307 |
| J07 | 0.425714286 | 0.480285626 | 0.005130569 |
| L01 | 0.257142857 | 0.308056504 | 0.0041558 |
| L02 | 0.001428571 | 0.002328469 | 1 |
| L03 | 0.002857143 | 0.004346476 | 0.76945788 |
| L04 | 0.007142857 | 0.014048432 | 0.177512192 |
| M01 | 0.797142857 | 0.885594536 | 6.27E-11 |
| M02 | 0.735714286 | 0.836619062 | 6.51E-11 |
| M03 | 0.207142857 | 0.283297113 | 8.03E-06 |
| M04 | 0.032857143 | 0.036168892 | 0.754331408 |
| M05 | 0.001428571 | 0.004036014 | 0.525366775 |
| M09 | 0.01 | 0.017618752 | 0.176407684 |
| N01 | 0.59 | 0.65406706 | 0.000621325 |
| N02 | 0.771428571 | 0.861223223 | 5.82E-10 |
| N03 | 0.077142857 | 0.085998137 | 0.445716975 |
| N04 | 0.005714286 | 0.010555728 | 0.331675912 |
| N05 | 0.238571429 | 0.289040671 | 0.003995814 |
| N06 | 0.501428571 | 0.516687364 | 0.437599719 |
| N07 | 0.135714286 | 0.153911829 | 0.214783879 |
| P01 | 0.142857143 | 0.167416951 | 0.09511496 |
| P02 | 0.144285714 | 0.147780192 | 0.82698237 |
| P03 | 0.021428571 | 0.030735796 | 0.17479058 |
| R01 | 0.94 | 0.971126979 | 2.37E-05 |
| R02 | 0.787142857 | 0.857187209 | 1.21E-06 |
| R03 | 0.73 | 0.818068923 | 2.71E-08 |
| R05 | 0.758571429 | 0.819698851 | 8.81E-05 |
| R06 | 0.667142857 | 0.727569078 | 0.000595928 |
| R07 | 0.354285714 | 0.394753182 | 0.035133826 |
| S01 | 0.965714286 | 0.985718721 | 0.000197459 |
| S02 | 0.891428571 | 0.944039118 | 1.42E-07 |
| S03 | 0.834285714 | 0.912682397 | 1.42E-10 |
| V01 | 0.152857143 | 0.200869295 | 0.001552486 |
| V03 | 0.312857143 | 0.358972369 | 0.013425923 |
| V04 | 0.032857143 | 0.046957467 | 0.095339334 |
